# Investigating the Influence of *ANTXR2* Gene Mutations on Protective Antigen Binding for Heightened Anthrax Resistance

**DOI:** 10.3390/genes15040426

**Published:** 2024-03-28

**Authors:** Chamalapura Ashwathama Archana, Yamini Sri Sekar, Kuralayanapalya Puttahonnappa Suresh, Saravanan Subramaniam, Ningegowda Sagar, Swati Rani, Jayashree Anandakumar, Rajan Kumar Pandey, Nagendra Nath Barman, Sharanagouda S. Patil

**Affiliations:** 1 ICAR-National Institute of Veterinary Epidemiology and Disease Informatics (NIVEDI), Bengaluru 560064, India; archanagowda7353@gmail.com (C.A.A.); yaminisekar0616@gmail.com (Y.S.S.); sagar.n.jnv@gmail.com (N.S.); swatirani221@gmail.com (S.R.); anandjayashree541@gmail.com (J.A.); ss.patil@icar.gov.in (S.S.P.); 2 ICAR-National Institute on Foot and Mouth Disease, Bhubaneswar 752050, India; s.subramaniam@icar.gov.in; 3 Department of Medical Biochemistry and Biophysics, Karolinska Institute, 17177 Solna, Sweden; rkpandey255@gmail.com; 4 College of Veterinary Science, Assam Agricultural University (AAU), Guwahati 781022, India; nnbarman@gmail.com

**Keywords:** *ANTXR2*, protective antigen, anthrax, molecular docking, single-nucleotide polymorphisms (SNPs)

## Abstract

*Bacillus anthracis* is the bacterium responsible for causing the zoonotic disease called anthrax. The disease presents itself in different forms like gastrointestinal, inhalation, and cutaneous. Bacterial spores are tremendously adaptable, can persist for extended periods and occasionally endanger human health. The Anthrax Toxin Receptor-2 (*ANTXR2*) gene acts as membrane receptor and facilitates the entry of the anthrax toxin into host cells. Additionally, mutations in the *ANTXR2* gene have been linked to various autoimmune diseases, including Hyaline Fibromatosis Syndrome (HFS), Ankylosing Spondylitis (AS), Juvenile Hyaline Fibromatosis (JHF), and Infantile Systemic Hyalinosis (ISH). This study delves into the genetic landscape of *ANTXR2*, aiming to comprehend its associations with diverse disorders, elucidate the impacts of its mutations, and pinpoint minimal non-pathogenic mutations capable of reducing the binding affinity of the *ANTXR2* gene with the protective antigen. Recognizing the pivotal role of single-nucleotide polymorphisms (SNPs) in shaping genetic diversity, we conducted computational analyses to discern highly deleterious and tolerated non-synonymous SNPs (nsSNPs) in the *ANTXR2* gene. The Mutpred2 server determined that the Arg465Trp alteration in the *ANTXR2* gene leads to altered DNA binding (*p* = 0.22) with a probability of a deleterious mutation of 0.808; notably, among the identified deleterious SNPs, rs368288611 (Arg465Trp) stands out due to its significant impact on altering the DNA-binding ability of *ANTXR2*. We propose these SNPs as potential candidates for hypertension linked to the *ANTXR2* gene, which is implicated in blood pressure regulation. Noteworthy among the tolerated substitutions is rs200536829 (Ala33Ser), recognized as less pathogenic; this highlights its potential as a valuable biomarker, potentially reducing side effects on the host while also reducing binding with the protective antigen protein. Investigating these SNPs holds the potential to correlate with several autoimmune disorders and mitigate the impact of anthrax disease in humans.

## 1. Introduction

Anthrax is an acute, rapidly progressing zoonotic disease caused by a Gram-positive, rod-shaped, spore-forming bacterium called *Bacillus anthracis* [1]. Anthrax is generally considered a rare disease, but it can have higher prevalence in certain regions. Worldwide, an estimated 20,000 to 100,000 cases of anthrax occur annually, mostly in poor rural areas [2]. In India, the states of Odisha, West Bengal, Andhra Pradesh, and Jharkhand have reported the highest number of anthrax outbreaks [3]. Specifically, Odisha has reported a maximum of 439 human anthrax cases since 2009, with the Koraput district contributing to 200 of these cases, accounting for 46% [3].

Furthermore, an outbreak in the Koraput district of Odisha recently resulted in the identification of 47 suspected cases of human anthrax. The outbreak started on 13 April 2023, and the epidemic curve indicated multiple point-source exposures [4]. Of these suspected cases, approximately 10 were confirmed as anthrax through RT-PCR testing [4]. *Bacillus anthracis* leads to the formation of spores that can remain dormant for many years in soil and begin to grow again and secrete toxins after gaining entry into susceptible hosts [5]. Bacterial spores cause severe breathing problems, shock, organ damage (sepsis) and inflammation around the brain and spinal cord, resulting in hemorrhagic meningitis and cell death [5]. The disease occurs in principal forms that include cutaneous (affecting the skin), inhalation (impacting the lungs), gastrointestinal (affecting the digestive system) as well as uncommon types [5].

*Bacillus anthracis* is composed of three virulence factors, protective antigen (PA), lethal factor (LF), and edema factor (EF), which are involved in the disease pathway. PA binds to cell receptors, undergoes proteolytic cleavage upon binding and forms complexes with LF and EF [6]. This allows the acidic endosomes, to trigger conformational changes in PA and achieves cytosolic entry through a pore in the endosomal membrane, facilitating the survival and germination of anthrax spores and disrupting host defense signaling pathways with the translocation of LF and EF into the cell cytoplasm. These complexes are endocytosis into target cells leading to anthrax symptoms [5]. The mechanism by which the anthrax toxin enters host cells is intricately linked to the interaction between the toxin and cell surface receptors, particularly Anthrax Toxin Receptor 2 (*ANTXR2*) and Anthrax Toxin Receptor 1 (ANTXR1) [6]. 

Both ANTXR1 and *ANTXR2*, classified as type I transmembrane proteins, share a structural framework characterized by a conserved extracellular ectodomain, a single-pass transmembrane domain and a cytosolic domain. The anthrax toxin functions primarily through the ectodomain, which is essential for binding to protective antigen (PA). In contrast, the transmembrane and cytosolic domains appear dispensable, as demonstrated by the unaffected activity of the anthrax toxin when the ectodomain is fused to a GPI (glycophosphatidylinositol)-linked structure on the membrane [7,8]. Although there is a 60% homology in the ectodomains of ANTXR1 and *ANTXR2*, notable differences in their binding affinities to protective antigen (PA) can be seen. Specifically, *ANTXR2* demonstrates a binding affinity that is at least a thousand times greater than that of ANTXR1 [9,10].

*ANTXR2*, a 55 kDa integrin-like type I transmembrane receptor, is also alternatively known as the capillary morphogenesis protein-2 (CMG-2) [11,12,13,14]. Situated on chromosome 4q21.21, the *ANTXR2* gene exhibits high conservation across various species and exists in four distinct isoforms generated through the process of alternative splicing [15,16]. Typically, *ANTXR2* comprises an extracellular ectodomain, including the von Willebrand factor type A (vWA) domain and an immunoglobulin-like domain, a 23-amino acid transmembrane segment, and a 148-amino acid cytosolic domain. This receptor holds significance in preserving the homeostasis of the extracellular matrix and plays a pivotal role in diverse physiological processes, such as vascular formation and angiogenesis [17]. 

*ANTXR2* stands out as the first determinant of virulence of B. anthracis, and the inactivation of this gene results in a highly robust protective effect, conferring complete resistance to both the anthrax toxin and the bacterium in the host [1]. Research has shown that variations in regulatory elements due to genetic factors can impact the transcription levels of the related genes [18,19]. It is reasonable to hypothesize the existence of such polymorphisms, particularly those located at transcription factor binding sites (TFBS) proximal to promoter or enhancer elements. These differences may be involved in controlling the expression of *ANTXR2*, consequently influencing anthrax toxin uptake and cellular susceptibility [20]. 

Understanding the genetic basis of intricate human diseases presents a significant challenge in modern genetics. Single-nucleotide polymorphisms (SNPs) play a vital role by providing important insights into the genetic differences underlying these illnesses and conditions [21]. The (SNPs) represent the most abundant form of human genetic sequence alterations, distributed widely across the entire genome of any organism [21,22]. A missense mutation, categorized as a type of nonsynonymous substitution (nsSNP), involves the replacement of one amino acid with another. This modification has the potential to yield a mutated protein with structural and functional changes, contributing to the onset of disease; nsSNPs are considered pivotal in shaping the functional diversity of proteins within the human population [23].

Anthrax, although often treatable with antibiotics, faces challenges due to the emergence of antibiotic resistance, especially against fluoroquinolones. Genetic mutations, such as those in the quinolone resistance-determining region (QRDR) and the GBAA0834 locus, contribute to resistance, highlighting the importance of ongoing research to understand and combat this issue effectively. In this case, studying the deleterious SNPs provides an alternative way [24]. nsSNPs are the single-nucleotide variations that affect the coding region of the protein and modify the mutated site-encoded amino acid, which may lead to a structural modification of the mutated protein, and may, thus, cause functional alteration [5]. These genetic variations are significant in many ways, including their role in preserving the structural integrity of cells and tissues [25]. Importantly, nsSNPs extend beyond their structural and regulatory functions. They also influence the protein’s functions in an array of biological processes such as signal transduction pathways linked to reactions to stimulants, hormones, and vision [21]. 

**Figure 1:**
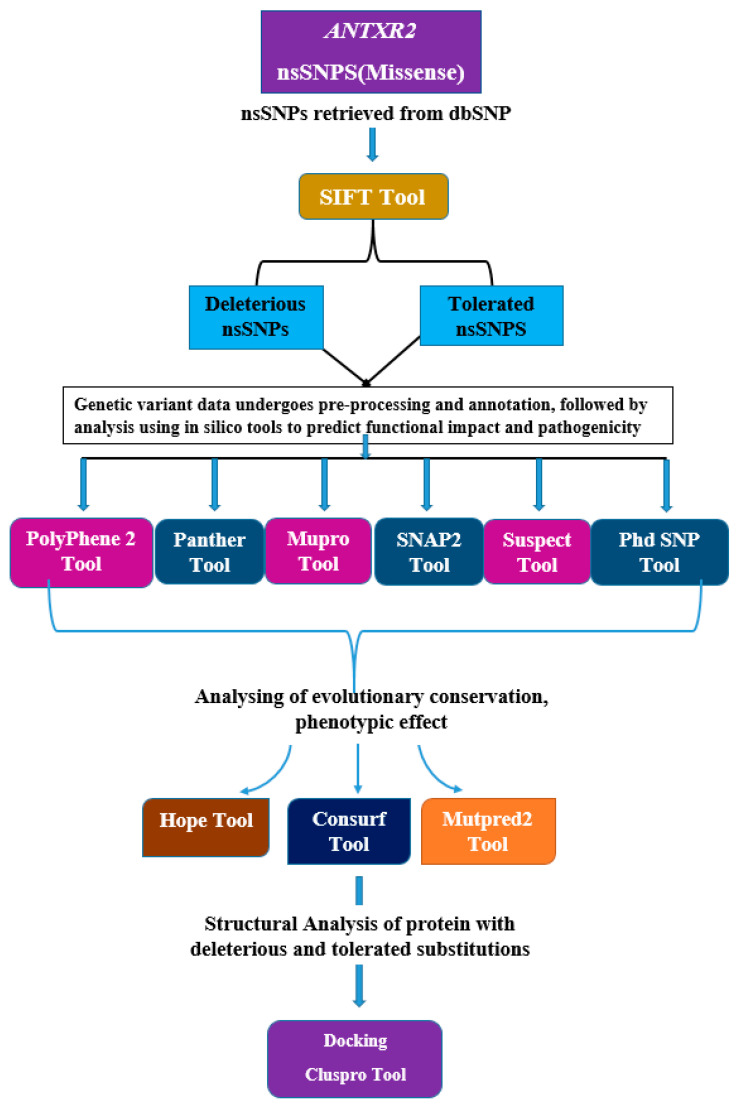
A flow chart showing the entire strategy and the tools that have been used in the study.

Identifying pathogenic or functionally associated SNPs in humans is a significant challenge for scientists [23,25]. In this study, because of their significant role in the pathogenesis of anthrax, the *ANTXR2* gene has been assessed for SNPs and examined for their impact on interaction with PA. Deleterious nsSNPs in the *ANTXR2* gene were identified using computational approaches and subsequently evaluated for potential effects. The methodology is depicted as a flow chart shown below (Figure 1). The study implicates certain SNPs in Hyaline Fibromatosis Syndrome and suggests others as potential candidates for hypertension related to the *ANTXR2* gene. Additionally, tolerated SNPs identified could serve as biomarkers for anthrax due to their impact on the binding affinity between the protective antigen and the *ANTXR2* gene.

## 2. Materials and Methods

### 2.1. Data Retrieval

*ANTXR2* gene protein sequences were retrieved in FASTA format using the UniProtKB database https://www.uniprot.org/ (accessed on 20 July 2023) and the National Centre for Biological Information (NCBI) https://www.ncbi.nlm.nih.gov/ (accessed on 20 July 2023). The Short Genetic Variation database (dbSNP) (https://www.ncbi.nlm.nih.gov/snp/) (accessed on 20 July 2023) was utilized to retrieve the gene’s SNPs. dbSNP is conceivable as a catalog of any short variations in the human nucleotide sequence. Only the nsSNPs (missense SNPs) were filtered and considered for further exploration. 

### 2.2. Characterization of Deleterious and Tolerated Non-Synonymous Variants

After retrieving the *ANTXR2* gene substitutions from the database, those substitutions were further analyzed to identify their tolerated and deleterious (harmful) nature, for which we employed the Sorting Intolerant from Tolerant (SIFT) tool, accessed at https://sift.bii.a-star.edu.sg/ (accessed on 9 August 2023) [26]. SIFT works by comparing the input sequence with multiple alignments of related sequences to predict a variation in protein function caused by the modification of the amino acid sequence [26,27]. Using dbSNP rsIDs (SIFT4G predictions), nsSNPs retrieved from the database were employed as input. Variants were classified as tolerated if their scores were ≥0.05 and as deleterious if their scores were ≤0.05 [28]. 

### 2.3. Predictions of Functional, Structural and Amino Acid Variations

To understand the functional and structural changes in the *ANTXR2* gene, the Protein Polymorphism Phenotyping v2.0 (PolyPhen-2) tool was utilized to identify tolerated and deleterious substitutions [29]. Accessed at http://genetics.bwh.harvard.edu/pph2/, (accessed on 15 August 2023), PolyPhen-2 assesses potential using a sequence-based approach [29]. Each amino acid substitution is assigned a qualitative prediction (probably damaging, potentially damaging, benign) [26]. The PolyPhen-2 position-specific independent count (PSIC) score, spanning from 0.0 to 1.0, serves as an indicator of the potential harm caused by a substitution, with elevated values indicating a greater likelihood of damage. The input included the Fasta sequence of the *ANTXR2* protein, along with details about the wild residue’s position in the protein and the mutated residue [29].

### 2.4. Exploring the Evolutionary Perspective in Protein Function

To delve into understanding the evolutionary conservation of the *ANTXR2* gene, we compare its substituted protein sequence with evolutionarily related protein sequences through the Protein Analysis Through Evolutionary Relationships (PANTHER) tool that was utilized. Accessed at http://www.pantherdb.org/tools/csnpScore.do (accessed on 20 August 2023), PANTHER involves comparing the protein sequence with an evolutionally related protein sequence. Substitution position-specific evolutionary conservation (subPSEC) scores are produced from the alignment of several proteins (stability and function) with their evolutionary relationships using substitution scores (subPSEC) of non-synonymous single-nucleotide polymorphisms (nsSNPs) [30]. Mutations with scores above 0.5 are labeled as harmful, whereas those below 0.5 are deemed less likely to cause harm, leveraging alignments of multiple proteins with evolutionary connections. The input included a list of tolerated and deleterious mutations, along with the protein sequence of the *ANTXR2* gene, with species selection limited to Homo sapiens [31].

### 2.5. Assessing Impact of Protein Stability and Sequence Information

To understand how mutations may affect the function and structure of the *ANTXR2* gene, the likelihood of a substitution reducing or not reducing the stability of the protein based on single-site amino acid mutations the MUpro tool was utilized [26]. The MUpro tool is based on machine learning methods such as Support Vector Machine and neural networks. Accessed at http://mupro.proteomics.ics.uci.edu/ (accessed on 24 August 2023), the MUpro tool assesses the confidence score to ascertain the impact of single-site mutations on protein stability, with a calculated score ranging from −1 to 1 indicating prediction reliability. Input can include the mutational position, original and substitution amino acids, and either the plain protein sequence or the structure of the protein [32,33].

### 2.6. Assessment of the Mutation Impact on Native Protein Characteristics

To gain further insight into the tolerated and deleterious effects of mutant variations through multiple-sequence and variant-features analysis, we utilized SNAP2. The impact of nsSNPs on secondary protein structure, pathogenicity, and solvent accessibility in both the *ANTXR2* gene and mutated proteins was analyzed by the tool SNAP2 (Screening for Nonacceptable Polymorphisms) [34]. Accessed at https://rostlab.org/services/snap2web/ (accessed on 11 September 2023), SNAP2 utilizes multiple sequence and variant features to distinguish between effect and neutral variants. The tool takes protein sequences in FASTA format as input and contrasts the solvent accessibility between the original and mutated proteins, generating a score ranging from −100 to +100, signifying a substantial effect prediction. This score provides a reliable assessment of the probability that a particular mutation will impact the inherent characteristics of the native protein [35]. The input comprised the Fasta sequence of the *ANTXR2* protein, accompanied by information regarding the position of the wild-type residue and the mutated residue. 

### 2.7. Predicting Phenotypic Outcomes of nsSNPs Using Integrated Sequence and Structural Information

We tend to understand the phenotypic effects caused by tolerated and deleterious mutations in the *ANTXR2* gene. Understanding phenotypic effects is important as it provides crucial insights into the functional consequences of genetic variations, aiding in disease diagnosis, prognosis, and treatment strategies. Therefore, in order to understand these effects, we employed SuSPect (Predict the phenotypic outcomes of non-synonymous single-nucleotide polymorphisms (nsSNPs)) tool [36]. Accessed at http://www.sbg.bio.ic.ac.uk/suspect/ (accessed on 16 September 2023), SuSPect utilizes a support vector machine (SVM) approach to predict phenotypic outcomes associated with nsSNPs. It generates a score table ranging from 0 to 100, which is color-coded to indicate the predicted deleteriousness of a variant (blue for neutral and red for disease-causing). A threshold of 50 is suggested to distinguish between variations classified as neutral and those that might have the potential to contribute to disease. The confidence level linked to these forecasts is indicated by values that are superior to or lower than 50 [36]. Only the amino acid sequence of the *ANTXR2* gene is used as the input.

### 2.8. Utilizing ConSurf for the Assessment of Proteins in Evolutionary Conservation

The ConSurf bioinformatics tool was utilized to integrate evolutionary conservation data with predictions of solvent accessibility to distinguish functional and structural residues within proteins [37]. Accessed at https://consurf.tau.ac.il (accessed on 21 September 2023), highly conserved residue is anticipated to be either functional or structural, contingent on its positioning on the protein surface or within its core [38]. The nsSNPs situated in these conserved regions are deemed considerably more detrimental to the protein compared to those at non-conserved sites [39,40,41]. ConSurf evaluates the conservation level of each residue in the target protein, categorizing it as variable, intermediate, or conserved, assigning a scale from 1 to 9 and categorizing it as variable (1–4), intermediate (5–6), or conserved (7–9) [32,42]. 

### 2.9. Structural and Phenotypic Analysis of Protein Mutations: Insights from HOPE and MutPred2 Tools

For examining the structural consequences of point mutations in protein sequences, the Have (y)Our Protein Explained (HOPE) server was utilized [43]. Accessed at https://www3.cmbi.umcn.nl/hope/ (accessed on 14 October 2023), HOPE generates a comprehensive report for each mutation, detailing its effects on the protein size, charge, bonding pattern, and interactions with other molecules. Our inputs encompassed UniProt sequences and individual SNPs [43]. 

Additionally, the phenotypic consequences of protein mutations were analyzed using the MutPred2 tool [44]. Accessed at http://mutpred.mutdb.org/ on 24 November 2023, MutPred utilizes neural networks to forecast the phenotypic effects of amino acid modifications. These outcomes may involve changes in protein stability, disruptions in protein structure, interference with macromolecular binding, and the possible removal of post-translational modification (PTM) sites, all contributing to substantial alterations in a protein’s phenotypic characteristics. For the analysis, the input included the protein’s FASTA sequence and the amino acid variations of interest, with the *p*-value threshold set to the default value of 0.05. Outputs with *p*-values < 0.05 and <0.01 were labeled as significant and very significant, respectively [32,45].

### 2.10. Molecular Docking

Lastly, the ClusPro server was utilized for protein–protein docking analysis, accessed at https://cluspro.org (accessed on 30 November 2023). To define the attraction, we specified the residues for both the ligand and the protein, using a “chain-residue” format with whitespace separation. In each docking experiment, this server generated the top 10 most likely docking poses. The representative docking poses, represented in [25], aim to elucidate potential interactions between protective antigen and *ANTXR2*.

## 3. Results

### 3.1. Data Retrieval and Sequence Analysis

Information pertaining to the Human *ANTXR2* gene and its protein sequence in FASTA format was gathered from the NCBI and UniportKB databases. SNPs within the *ANTXR2* gene were obtained from the dbSNP database. A total of 60,997 SNPs were identified, with the present study specifically concentrating on 436 SNPs classified as non-synonymous. All 436 SNPs obtained from databases have been included in the Supplementary Table S1. 

### 3.2. Identification and Prediction of Both Tolerated and Deleterious SNPs

The dbSNP database’s 436 SNPs were subjected to computational analysis employing various tools, including SIFT, PolyPhen-2, Suspect, Mupro, Panther, and PhD SNP. Of the non-synonymous variants screened using the SIFT technique, 23 were found to be the most deleterious, while 21 were found to be tolerated. The deleterious and tolerated SNPs were then segregated and individually assessed. Among the deleterious SNPs, PolyPhen-2 identified 15 as probably damaging, 1 as possibly damaging, and 7 as benign. A neural network-based classification program called SNAP2 was then used to predict changes in the secondary structure caused by nsSNPs. This method predicted 17 variants with a substantial effect, while 6 variants were found to be neutral. The SuSPect tool identified 15 substitutions with a score above 50 as disease-associated and 8 with a score below 50 as neutral. Analysis of the substitutions’ impact on protein stability using MUpro revealed 20 substitutions associated with decreased stability of the protein, while 3 were shown to be associated with increased stability. PANTHER was utilized to calculate the likelihood of harmful nsSNPs by substituting specific-position evolutionary conservation, which showed 8 substitutions as probably benign and 15 as possibly damaging. PhD-SNP identified 13 SNPs as disease-associated polymorphisms and 10 as neutrals. Regarding the tolerated SNPs, PolyPhen-2 found 2 probably damaging, 1 possibly damaging, 2 benign, and the remainder as errors. SNAP2 revealed 3 variations as disease-associated, 4 as neutral, and 15 errors. SuSPect identified 1 substitution with a score above 50 as disease-associated and 5 with a score below 50 as neutral. PANTHER revealed probably benign in 3 cases, possibly damaging in 3, and 15 neutrals. PhD-SNP resulted in all neutrals (Table 1 and Table 2).

### 3.3. Mutational Effect on the Structural and Phenotypic Characteristics of Protein

The impact of amino acid substitutions on the chemical and physical properties, hydrophobicity, spatial structure, and function of proteins was predicted using the HOPE program. The analyses indicated a shift in charge from neutral to negative for glycine at position 450 (Gly450Asp) and glycine at position 150 (Gly105Asp). Additionally, there was an observed change from neutral to positive for glycine at position 300 (Gly300Arg) and leucine at position 329 (Leu329Arg). Furthermore, a subsequent alteration from positive to neutral was noted in the case of arginine at position 465 (Arg465Trp). Furthermore, mutants Arg465Trp and Tyr381Cys increased hydrophobicity, whereas mutants Gly300Arg, Gly105Asp, Ile189Thr, and Leu329Arg decreased hydrophobicity.

The analysis of tolerated variants based on HOPE revealed a change in charge from negative to positive at position 194 with the substitution of glutamic acid by lysine (Glu194Lys). Additionally, mutants Thr258Ala and Ala33Ser increased hydrophobicity, whereas mutants Ala70Val, Ala280Pro, Glu194Lys, Leu169Phe, and Val270Ile decreased hydrophobicity. Differences in hydrophobicity and hydrogen-bonding connections between adjacent residues may be disrupted by differences in size between wild-type and mutant residues and the overall protein framework (Table 3).

Upon further analysis by the MutPred2 server, it was found that 7 out of the 9 nsSNPs scored higher than 0.8, indicating their high pathogenic properties. Notably, Tyr381Cys exhibited a loss of phosphorylation, Gly300Arg showed a loss of GPI-anchor amidation, while Gly300Arg, Gly105Asp, Ile189Thr, Leu329Arg, and Arg465Trp displayed alterations in transmembrane protein characteristics. Ala454Val, Gly450Asp, and Arg465Trp exhibited changes in the disordered interface. Ile189Thr and Arg465Trp showed modified metal binding. Additionally, Ile189Thr displayed altered stability and Arg465Trp showed changes in DNA binding. Simultaneously the 7 tolerated substitutions scored lower than 0.8, indicating their low pathogenic properties (Table 4).

### 3.4. Conservation and Evolutionary Preservation Patterns of nsSNPs

As indicated by the ConSurf output, among the 9 nsSNPs, 7 variants (Ala454Val, Gly300Arg, Gly105Asp, Ile189Thr, Arg465Trp, Trp341Leu, and Tyr381Cys) are situated within highly conserved regions, underscoring their significant preservation across evolutionary time. In addition, 2 variants (Gly450Asp and Leu329Arg) are positioned in moderately conserved regions, indicating a considerable but less stringent degree of conservation. Conversely, in the case of tolerated nsSNPs, among the 7 identified variants, 2 (Ala70Val and Ala280Pro) occur in regions with high conservation, suggesting that these specific amino acid substitutions are maintained across a variety of species. On the other hand, the remaining 5 variants (Ala33Ser, Glu194Lys, Leu169Phe, Thr258Ala, and Val270Ile) are situated in moderately conserved regions, implying a noticeable but not as stringent level of conservation in these regions (Table 5).

### 3.5. Impact of (nsSNPs) on ANTXR2 Gene Binding Affinity with Protective Antigen

The binding affinity of the *ANTXR2* gene and protective antigen is −1593.2. The docking analysis confirmed that seven out of nine nsSNPs (Ala454Val, Gly450Asp, Gly300Arg, Leu329Arg, Arg465Trp, Trp341Leu and Tyr381Cys) lowered the binding affinity of the PA protein by −882.5. Following Ile189Thr and Gly105Asp resulted in −883.1 and −883.4, respectively. At the same time, among the 7 tolerated variants, 5 (Ala33Ser, Ala70Val, Ala280Pro, Thr258Ala, Val270Ile resulted in −882.5 binding affinity with PA protein. Following Glu194Lys and Leu169Phe resulted in −806.8 and −881.5 binding affinity, respectively. The *ANTXR2* protein’s functional activity can be considerably impacted by the aforementioned variations, according to the molecular docking analysis (Table 6).

## 4. Discussion

The protective antigen (PA) of the Bacillus anthracis toxin enters cells by binding to *ANTXR2* receptors which exhibit a strong affinity for PA and can facilitate toxicity [46,47]. In addition to playing a significant role in the pathogenesis of anthrax, *ANTXR2* has been shown to play a role in other conditions as well. The four mutations, rs137852902, rs137852905, rs137852903, rs137852901, identified among the deleterious SNPs are associated with Hyaline Fibromatosis Syndrome. This autosomal recessive condition is characterized by the accumulation of hyalinizing fibrosis [48,49]. *ANTXR2* mutations are connected to the development of Ankylosing Spondylitis (AS), a chronic inflammatory condition affecting the spine and sacroiliac joints. This disease is characterized by arthritis and enthesitis in both the spine and peripheral joints [50]. *ANTXR2* mutations in a family were linked to the occurrence of Juvenile Hyaline Fibromatosis (JHF). This uncommon autosomal recessive disorder, of unknown prevalence, is characterized by the atypical development of hyalinized fibrous tissue, typically manifesting in the skin, mucosa, bone, and frequently affecting internal organs [51]. Infantile Systemic Hyalinosis (ISH) is a rare genetic condition that is autosomal-recessive and detected by dermal and subcutaneous fibromatosis, joint contractures, and bone deformities. Typically manifesting at birth, this condition unfortunately leads to mortality during infancy. The underlying cause of ISH lies in mutations within the *ANTXR2* [52]. *ANTXR2* appears crucial in blood pressure regulation, potentially through angiogenesis or vascular–smooth muscle contraction pathways [53]. The objective of the study was to identify deleterious SNPs that disrupt the function of the *ANTXR2* gene and conversely to pinpoint tolerated SNPs that diminish the binding affinity of PA with the *ANTXR2* gene. 

Blood cells taken from hunter-gatherers were reported to have somewhat greater levels of *ANTXR2* expression than surrounding agricultural groups in Africa [53]. This finding may be compatible with agricultural populations having a higher chance of contracting anthrax disease. According to another study, the *ANTXR2* locus has a complicated evolutionary history, with selection acting on several alleles to continuously influence *ANTXR2* expression variations in human populations [54]. This finding may be compatible with agricultural populations having a higher chance of contracting anthrax disease. According to another study, the *ANTXR2* locus has a complicated evolutionary history, with selection acting on several alleles to continuously influence *ANTXR2* expression variations in human populations [55]. Therefore, it is imperative to explore the repercussions of detrimental nsSNPs in *ANTXR2* and their possible links to different illnesses. Furthermore, it is necessary to find beneficial SNPs that could serve as potential biomarkers against anthrax disease by limiting their binding affinity with PA. In this context, we conducted a computational analysis to identify the most detrimental and non-harmful nsSNPs and analyze their impact on the *ANTXR2* protein’s structure and functionality.

To enhance the reliability of predicting harmful non-synonymous SNPs (nsSNPs), a diverse array of techniques was employed for initial screening. Among them tools like SIFT and Mutation Assessor relied on factors like sequence homology and amino acid physical properties for their predictions, while others like SNAP2 and PolyPhen2 utilized machine learning to forecast the structural and functional consequences of alterations. Additionally, SNP&GO, SuSPect, and PANTHER were incorporated in the analysis to assess whether the discovered polymorphisms were connected to pathogenicity. Recognizing the significance of protein stability in structural and functional activity [40], we utilized MUpro to pinpoint deleterious nsSNPs that could potentially impact the stability of the *ANTXR2* protein.

In total, 9 non-synonymous single-nucleotide polymorphisms (nsSNPs) were identified as highly detrimental, as they were concurrently predicted to be high-risk by the SNP prediction algorithms employed in this study. Additionally, 7 nsSNPs were consistently deemed as tolerated by all the utilized tools. The highly pathogenic nsSNPs identified through this approach include: rs372562244, rs137852902, rs190198202, rs369528902, rs137852905, rs137852903, rs368288611, rs77105256, rs137852901. Conversely, the non-harmful SNPs encompass rs200536829, rs370619047, rs12647691, rs376076187, rs374723881, rs368740456, rs113707133. The Project Hope server analysis identified 5 nsSNPs (A454VAla454Val, Gly450Asp, Gly300Arg, Gly105Asp, and Leu329Arg) among the 11 considered to be highly risky, indicating a detrimental impact on the protein’s structure. Additionally, in the tolerated case, one mutation (Thr258Ala) was observed to affect the protein’s structure, while the remaining substitution of the protein exhibited no variability in structure.

According to MutPred2 results, Arg465Trp emerges as the most deleterious substitution, displaying alterations in the ordered and disordered interface, transmembrane protein characteristics, DNA binding, and metal binding. Conversely, tolerated substitutions exhibit a less pathogenic effect, qualifying them as potential biomarker candidates. The ConSurf analysis revealed that 7 (Ala454Val, Gly300Arg, Gly105Asp, Ile189Thr, Arg465Trp, Trp341Leu, Tyr381Cys) nsSNPs reside within highly conserved regions. Among the tolerated nsSNPs, Ala33Ser was identified in a variable region, while Leu169Phe, Thr258Ala, and Val270Ile were situated in moderately variable regions, suggesting their relative safety in terms of structural impact.

The ClusPro server provides a binding affinity scale to assess the strength of protein–protein interactions. The docking analysis confirmed that 9 highly deleterious nsSNPs markedly diminished the binding affinity with the PA protein in contrast to the residues of the wild-type. Similarly, within the tolerated substitutions, 7 alterations also exhibited a decrease in binding affinity with the PA protein. It is intriguing that 7 SNPs, which differ in terms of the position, size, and charge of the amino acid, collectively decreased the original binding affinity from −1593.2 to −882.5. This reduction may result from cumulative disruptions in key interaction sites or structural features critical for the stability of the *ANTXR2* protein complex.

Highly detrimental substitutions are those that significantly alter the protein’s structure and its function. These substitutions may disrupt critical protein–protein interactions, interfere with enzymatic activity, or destabilize the protein’s overall structure. As a result, they are predicted to have a detrimental impact on the protein’s ability to carry out its biological functions effectively. On the other hand, tolerated substitutions are those that have minimal or no adverse effects on the protein’s structure or function. These substitutions may occur in regions of the protein that are less critical for its overall stability or function. While they may result in minor changes to the protein, they are generally well-tolerated and do not significantly impair its functionality. The mutation rs12647691 being shown as the less pathogenic in 2 tools (SIFT and Suspect), with information not available for the remaining tools, is found to be associated with anthrax toxin susceptibility. Along with rs13140055 and rs80314910, it modulates *ANTXR2* promoter activity, revealing a significant correlation between human genetic variations and anthrax toxin sensitivity [20].

If both highly deleterious and tolerated substitutions result in a reduction in the binding affinity with the PA protein, it suggests that these genetic variations could compromise the protein’s ability to effectively interact with the PA protein. This could have significant implications for disease susceptibility or response to treatment, as the effectiveness of the protein in its intended function may be impaired. Additionally, the use of computational methods like docking analysis enables the identification of these pathogenic nsSNPs without the need for extensive laboratory experimentation.

## 5. Conclusions

In addition to modulating pathogenesis of *B. anthracis*, mutations in the *ANTXR2* gene play a role in various disorders, emphasizing the crucial need for understanding their implications. Our in-depth computational analysis has successfully identified both highly harmful and tolerated non-synonymous single-nucleotide polymorphisms (nsSNPs), providing insights into potential biomarkers and disease associations. Notably, we pinpointed some particularly damaging SNPs, such as rs372562244, rs137852902, rs190198202, rs369528902, rs137852905, rs137852903, rs368288611, rs77105256, and rs137852901. Among them, rs368288611 (Arg465Trp) stands out for its severe impact on altering the DNA-binding ability of the *ANTXR2* gene. The study identified four mutations (rs137852902, rs137852905, rs137852903, and rs137852901) among the deleterious SNPs linked to Hyaline Fibromatosis Syndrome. Other substitutions (rs372562244, rs190198202, rs369528902, rs368288611, rs77105256) remain unexplored, lacking clinical characterization. We propose these SNPs as potential candidates for hypertension linked to the *ANTXR2* gene, which is implicated in blood pressure regulation, possibly through angiogenesis or vascular contraction pathways. On the other hand, we identified tolerated substitutions like rs200536829, rs370619047, rs12647691, rs376076187, rs374723881, rs368740456, and rs113707133. Noteworthy among them is rs200536829 (Ala33Ser), recognized as less pathogenic. These tolerated and less harmful substitutions could serve as valuable biomarkers, potentially reducing side effects on the host while also lessening binding with the protective antigen protein. Given the association of deleterious SNPs with diverse diseases, utilizing tolerated SNPs identified in the study—shown to reduce the binding affinity between the protective antigen and *ANTXR2* gene—may prove effective as a biomarker against anthrax. This opens a promising avenue for preventing anthrax in humans. However, it is crucial to emphasize that experimental validation is essential to confirm the findings of this study.

## Figures and Tables

**Table 1:** Comprehensive analysis of single-nucleotide polymorphisms (SNPs) using various tools to identify deleterious and tolerated variants, assessing their impact on protein stability.

Sl.No	SNP ID	Amino Acid Variant	Single Nucleotide Variation	SIFT		PolyPhen 2	Panther
				Score	Prediction		
**1**	rs372562244	Ala454Val	G/A	0	Deleterious	Probably damaging	Probably damaging
**2**	rs137852902	Gly105Asp	C/T	0.003	Deleterious	Probably damaging	Probably damaging
**3**	rs190198202	Gly300Arg	C/T	0.003	Deleterious	Probably damaging	Probably damaging
**4**	rs369528902	Gly450Asp	C/T	0	Deleterious	Probably damaging	Probably damaging
**5**	rs137852905	Ile189Thr	A/G	0.001	Deleterious	Probably damaging	Probably damaging
**6**	rs137852903	Leu329Arg	A/C	0.002	Deleterious	Probably damaging	Probably damaging
**7**	rs368288611	Arg465Trp	G/A	0.001	Deleterious	Probably damaging	Probably damaging
**8**	rs77105256	Trp341Leu	C/A	0	Deleterious	Probably damaging	Probably damaging
**9**	rs137852901	Tyr381Cys	T/C	0.001	Deleterious	Probably damaging	Probably damaging
**Tolerated**							
**1**	**rs200536829**	Ala33Ser	C/A	0.665	Tolerated	Probably damaging	Probably benign
**2**	**rs370619047**	Ala70Val	G/A	0.368	Tolerated	Probably damaging	Possibly damaging
**3**	**rs12647691**	Ala280Pro	C/G	1	Tolerated	Probably damaging	Possibly damaging
**4**	**rs376076187**	Glu194Lys	C/T	0.174	Tolerated	Probably damaging	Probably benign
**5**	**rs374723881**	Leu169Phe	G/A	0.186	Tolerated	Probably damaging	Possibly damaging
**6**	**rs368740456**	Thr258Ala	T/C	0.538	Tolerated	Probably damaging	Probably benign
**7**	**rs113707133**	Val270Ile	C/T	0.862	Tolerated	Probably damaging	Probably benign

**Table 2:** Details of genetic variations in the *ANTXR2* gene predicted as high-risk SNPs out of 486 nsSNPs.

Sl.No	SNP ID	Amino Acid Variant	PhD SNP		Mu Pro Prediction		SNAP2		Suspect
			Prediction	Score		Score	Score	Prediction	
**1**	**rs372562244**	Ala454Val	Diseases	0.639	Decrease stability	−0.62145	59	Effect	64
**2**	**rs137852902**	Gly105Asp	Diseases	0.645	Decrease stability	−0.8272	47	Effect	55
**3**	**rs190198202**	Gly300Arg	Diseases	0.781	Decrease stability	−1.3046	83	Effect	87
**4**	**rs369528902**	Gly450Asp	Diseases	0.828	Decrease stability	−0.999	80	Effect	53
**5**	**rs137852905**	Ile189Thr	Diseases	0.654	Decrease stability	−2.3902	61	Effect	86
**6**	**rs137852903**	Leu329Arg	Diseases	0.842	Decrease stability	−2.3037	49	Effect	65
**7**	**rs368288611**	Arg465Trp	Diseases	0.708	Decrease stability	−0.8297	87	Effect	72
**8**	**rs77105256**	Trp341Leu	NA	NA	Decrease stability	−0.17876	81	Effect	80
**9**	**rs137852901**	Tyr381Cys	Diseases	0.842	Decrease stability	−0.76939	57	Effect	33
**Tolerated**									
**1**	**rs200536829**	Ala33Ser	Neutral	0.074	Decrease stability	−0.8690	94	Neutral	NA
**2**	**rs370619047**	Ala70Val	Neutral	0.149	Decrease stability	−0.2488	98	Neutral	21
**3**	**rs12647691**	Ala280Pro	NA	NA	NA	NA	28	Neutral	75
**4**	**rs376076187**	Glu194Lys	Neutral	0.277	Decrease stability	−1.0843	6	Neutral	21
**5**	**rs374723881**	Leu169Phe	Neutral	0.441	Decrease stability	−1.2402	63	Neutral	13
**6**	**rs368740456**	Thr258Ala	Neutral	0.295	Decrease stability	−0.9580	53	Neutral	46
**7**	**rs113707133**	Val270Ile	Neutral	0.032	Increase Stability	0.0840	81	Neutral	39

NA-Not Available.

**Table 3:** Interpretation of the impact of amino acid variants: predicting the impact on chemical and physical qualities, hydrophobicity, spatial structure, protein stability and protein function by HOPE.

Sl.No	Residue	Change of Size	Change of Charge	Change of Hydrophobicity	Properties
1	Ala454Val	W < M	NA	NA	Although the wild-type residue is significantly conserved, there have been instances of different residue types at this position. No occurrences of the mutant residue or a similar type were identified in other homologous sequences, indicating potential protein damage according to conservation scores. The increased size of the mutant residue could lead to structural irregularities.
2	Gly450Asp	W < M	Neutral to Negative	Wild type residue more hydrophobicity than mutant	Amino acids exhibit unique size, with mutants often being larger and experiencing shifts in charge. The wild-type residue tends to be more hydrophobic.
3	Gly300Arg	W < M	Neutral to Positive	Wild type residue more hydrophobicity than mutant	A charge difference exists between wild-type and mutant amino acids. The mutation introduces a charge, potentially causing repulsion of ligands or residue with the same charge. Wild-type and mutant amino acids differ in size. The mutant residue’s increased size may lead to bumps.
4	Gly105Asp	W < M	Neutral to Negative	Wild type residue more hydrophobicity than mutant	The wild-type residue is neutral; the mutant residue is negative. The mutant residue, along with similar types, is not found at this position in other homologous sequences. Conservation scores suggest probable damage to the protein due to this mutation.
5	Ile189Thr	W > M	NA	Wild type residue more hydrophobicity than mutant	NA
6	Leu329Arg	W < M	Neutral to Positive	Wild type residue more hydrophobicity than mutant	The wild-type residue is neutral; the mutant residue is positive.
7	Arg465Trp	W < M	Wild Positive and mutant neutral	Mutant type residue more hydrophobicity than wild	The wild-type residue is positive; the mutant residue is neutral.
8	Trp341Leu	W > M	Positive to Neutral	NA	NA
9	Tyr381Cys	W > M	NA	Mutant type residue more hydrophobicity than wild	NA
**Tolerated**					
1	Ala33Ser	W > M	NA	Wild type more hydrophobic than mutant	NA
2	Ala70Val	W > M	NA	NA	NA
3	Ala280Pro	NA	NA	NA	The mutated residue is situated in a vital protein domain, interacting with another crucial domain essential for the protein’s activity.
4	Glu194Lys	W > M	Wild negative to mutant positive	NA	The mutation has the potential to disrupt their interaction, potentially affecting the overall function of the protein.
5	Leu169Phe	W > M	NA	NA	The mutant residue is one of the types of homologous sequences that have been observed at this position, suggesting that this mutation is likely non-damaging to the protein at this location.
6	Thr258Ala	W < M	NA	Mutant type has more hydrophobicity than wild	The mutant residue is found among observed types in homologous sequences, typically indicating non-damage to the protein. However, in this instance, it has been established that the mutation is deleterious.
7	Val270Ile	W < M	NA	NA	The mutant residue is commonly observed in homologous sequences, implying potential harmlessness. It has been confirmed in this case to be deleterious to the protein’s structure and function.

NA-Not Available.

**Table 4:** MutPred2 analysis reveals high pathogenic properties in selected nsSNPs.

Sl.No	Mutation	Probability of Deleterious Mutation	Structural and Functional Properties
**1**	Ala454Val	0.701	Altered Disordered interface (*p* = 0.30) Gain of proteolytic cleavage at D453 (*p* = 0.12)
**2**	Gly450Asp	0.896	Altered Disordered interface (*p* = 0.32) Gain of Intrinsic Disordered (*p* = 0.30) Gain of proteolytic cleavage at D453 (*p* = 0.14)
**3**	Gly300Arg	0.802	Altered Transmembrane protein (*p* = 0.27) Gain of ADP-ribosylation at G300 (*p* = 0.21) Loss of GPI-anchor amidation at N298 (*p* = 0.02)
**4**	Gly105Asp	0.573	Gain of Helix (*p* = 0.27) Altered Transmembrane protein (*p* = 0.17) Gain of Ubiquitylation at K104 (*p* = 0.16)
**5**	Ile189Thr	0.831	Gain of Relative solvent accessibility (*p* = 0.27) Altered Stability (*p* = 0.22) Altered Metal binding (*p* = 0.21) Altered Transmembrane protein (*p* = 0.13)
**6**	Leu329Arg	0.913	Altered Transmembrane protein (*p* = 0.27) Altered Signal peptide (*p* = 0.12)
**7**	Arg465Trp	0.808	Altered Ordered interface (*p* = 0.35) Altered Transmembrane protein (*p* = 0.32) Gain of Helix (*p* = 0.27) Altered DNA binding (*p* = 0.22) Gain of Allosteric site at R465 (*p* = 0.21) Altered Disordered interface (*p* = 0.18) Altered Metal binding (*p* = 0.17) Gain of Pyrrolidone carboxylic acid at Q462 (*p* = 0.08)
**8**	Trp341Leu	0.846	Altered Ordered interface (*p* = 0.28)
**9**	Tyr381Cys	0.914	Loss of Phosphorylation at Y381 (*p* = 0.39) Altered Disordered interface (*p* = 0.27) Gain of Methylation at R384 (*p* = 0.13) Gain of Proteolytic cleavage at D377 (*p* = 0.11)
**Tolerated**			
1	Ala33Ser	0.137	NA
2	Ala70Val	0.543	Altered Transmembrane protein (*p* = 0.30)
3	Ala280Pro	NA	NA
4	Glu194Lys	0.657	Altered Transmembrane protein (*p* = 0.34) Gain of Relative Solvent accessibility (*p* = 0.29) Loss of Loop (*p* = 0.28) Altered Metal binding (*p* = 0.21)
5	Leu169Phe	0.193	NA
6	Thr258Ala	0.242	NA
7	Val270Ile	0.076	NA

NA-Not Available.

**Table 5:** ConSurf Analysis of nsSNPs showing evolutionary conservation profile of amino acid in *ANTXR2* gene and significance in protein regions.

SL.NO	Substitution	Scores	ConSurf
1	Ala454Val	9	Conserved
2	Gly450Asp	5	Average
3	Gly300Arg	8	Conserved
4	Gly105Asp	9	Conserved
5	Ile189Thr	8	Conserved
6	Leu329Arg	4	Average
7	Arg465Trp	9	Conserved
8	Trp341Leu	9	Conserved
9	Tyr381Cys	9	Conserved
**Tolerated**			
1	Ala33Ser	1	Variables
2	Ala70Val	7	Conserved
3	Ala280Pro	7	Conserved
4	Glu194Lys	5	Average
5	Leu169Phe	4	Average
6	Thr258Ala	4	Average
7	Val270Ile	4	Average

**Table 6:** Molecular Docking Analysis of *ANTXR2* Gene Variants: Impact on Binding Affinity with Protective Antigen and Implications for Functional Activity.

Sl.No	Substitution	Binding Affinity
	Wild Type Deleterious	−1593.2
1	Ala454Val	−882.5
2	Gly450Asp	−882.5
3	Gly300Arg	−882.5
4	Gly105Asp	−883.4
5	Ile189Thr	−883.1
6	Leu329Arg	−882.5
7	Arg465Trp	−882.5
8	Trp341Leu	−882.5
9	Tyr381Cys	−882.5
**Tolerated**		
1	Ala33Ser	−882.5
2	Ala70Val	−882.5
3	Ala280Pro	−882.5
4	Glu194Lys	−806.8
5	Leu169Phe	−881.5
6	Thr258Ala	−882.5
7	Val270Ile	−882.5

## Data Availability

The data that support the findings of this study are available on NCBI dbSNP (https://www.ncbi.nlm.nih.gov/snp/).

## Supplementary Materials

The following supporting information can be downloaded at: https://www.mdpi.com/article/10.3390/genes15040426/s1, Table S1: 436snps retrieved from dbSNP database.
